# A brief intervention targeting primary-care physician prescribing of pharmacotherapies for alcohol dependence: can it impact prescribing behavior and reduce hospital inpatient admissions?

**DOI:** 10.1186/1940-0640-7-S1-A75

**Published:** 2012-10-09

**Authors:** Anthony Shakeshaft

**Affiliations:** 1 National Drug and Alcohol Research Center, University of New South Wales, Sydney, Australia

## 

Increasing the use of pharmacotherapies for alcohol dependence has the potential to improve patient outcomes and reduce health-care costs by reducing hospital admissions. This randomized controlled trial (RCT) evaluated the cost-effectiveness of tailored mailed feedback on general practitioner (GP) prescribing for alcohol dependence and alcohol-related hospital admissions. General practitioners (N = 115) in 10 communities randomized to the experimental arm of the Alcohol Action in Rural Communities (AARC) project received tailored mailed feedback on their prescribing of acamprosate and naltrexone. Segmented regression analysis examined the impact of the intervention relative to GPs’ prescribing and inpatient hospital admissions for alcohol dependence in those communities. Incremental cost-effectiveness ratios were estimated to compare costs per additional prescription written and costs per inpatient admission averted. Trend analysis showed GPs significantly increased their prescribing of acamprosate (ß = 0.24; 95% confidence interval [CI], 0.13-0.35) and significantly decreased their prescribing of naltrexone (ß = 0.12; 95% CI, 0.13-0.35). Rates of alcohol-related inpatient admissions for alcohol dependence decreased significantly in the experimental group compared with the control group (ß = 0.98; 95% CI, 1.80-0.16). Similar to evidence showing SBI can improve patient outcomes, this study showed mailed tailored feedback to GPs achieved cost-effective increases in their prescribing of acamprosate, with a subsequent and plausibly causal reduction in inpatient hospital admissions for alcohol dependence. Demonstrating the capacity of brief intervention in primary-care settings to reduce demand for tertiary care services appears to be a promising direction for the SBI field. A large-scale RCT of the cost benefit of tailored feedback to GPs appears warranted.

